# The Role of Microtubules in Pancreatic Cancer: Therapeutic Progress

**DOI:** 10.3389/fonc.2021.640863

**Published:** 2021-05-21

**Authors:** Mugahed Abdullah Hasan Albahde, Bulat Abdrakhimov, Guo-Qi Li, Xiaohu Zhou, Dongkai Zhou, Hao Xu, Huixiao Qian, Weilin Wang

**Affiliations:** ^1^Department of Hepatobiliary and Pancreatic Surgery, The Second Affiliated Hospital, School of Medicine, Zhejiang University, Hangzhou, China; ^2^Key Laboratory of Precision Diagnosis and Treatment for Hepatobiliary and Pancreatic Tumor of Zhejiang Province, Hangzhou, China; ^3^Research Center of Diagnosis and Treatment Technology for Hepatocellular Carcinoma of Zhejiang Province, Hangzhou, China; ^4^Clinical Medicine Innovation Center of Precision Diagnosis and Treatment for Hepatobiliary and Pancreatic Disease of Zhejiang University, Hangzhou, China; ^5^Department of Cardiovascular Surgery, Renmin Hospital of Wuhan University, Wuhan, China; ^6^Clinical Research Center of Hepatobiliary and Pancreatic Diseases of Zhejiang Province, Hangzhou, China

**Keywords:** pancreatic ductal adenocarcinoma, microtubule-targeting agents, microtubules, pancreatic cancer, drugs

## Abstract

Pancreatic cancer has an extremely low prognosis, which is attributable to its high aggressiveness, invasiveness, late diagnosis, and lack of effective therapies. Among all the drugs joining the fight against this type of cancer, microtubule-targeting agents are considered to be the most promising. They inhibit cancer cells although through different mechanisms such as blocking cell division, apoptosis induction, etc. Hereby, we review the functions of microtubule cytoskeletal proteins in tumor cells and comprehensively examine the effects of microtubule-targeting agents on pancreatic carcinoma.

## Overview of Pancreatic Ductal Adenocarcinoma

Pancreatic ductal adenocarcinoma (PDAC) is a malignant tumor and the fourth leading cause of tumor-related death in the world. It has a 5-year survival rate of 6% to 7% (1–3). PDAC is the most common type of pancreatic cancer (PC), accounting for more than 90% of this malignancy (4). In 2016, in the United States alone, the number of new cases and deaths were estimated to be 53,000 and 42,000, respectively (5). At present, PDAC is the 12th most diagnosed malignancy worldwide. The incidence of PDAC is 8 to 12.5/100000 among males and 6 to 7/100,000 among females, but this rate continues to increase (6). Low survivability in PDAC is due to its high aggressiveness and invasiveness as well as lack of effective and efficient diagnostic tools and therapies. Radical surgery is considered to be the first-line treatment of early-stage PDAC (7). However, even upon diagnosing PDAC at an early stage, only 9.7% of patients can receive surgical treatment. For advanced-stage PDAC patients (diagnosed in 85% of cases), chemotherapy is the only treatment option (5, 8). At this rate, PDAC is expected to become the 2nd most common cause of mortality among all the malignancies by the end of the decade (9).

Most PDAC patients have a very poor response rate to chemotherapy (10). High systemic drug resistance is a result of dense stroma that drugs cannot effectively penetrate and complex cellular processes, such as abnormal gene expression, gene mutation, abnormal activation or inhibition of cell signaling pathways, etc. (11–13). More effective drugs and treatment regimens targeted on PDAC are needed to improve its clinical outcome (14). Research toward increasing the survival rate has been carried out for many years. The investigations were focused on exploring prognostic markers and novel mechanisms of PDAC carcinogenesis. Despite the advancement in PDAC research, the difference in the median overall survival for PDAC between 1986 and 2016 was found to be minimal (15). Researchers require the illustration of novel molecular targets and alternative approaches to PDAC therapy. This review focuses on the functions of microtubule cytoskeletal proteins in tumor cells and comprehensively examines the effects of microtubule-targeting agents on PDAC.

## Overview of Microtubules

Microtubules are one of the three main components of the cytoskeleton and are involved in a variety of essential cellular processes and functions. Microtubules are tube-shaped protein polymers approximately 25 nm in diameter formed by the combination of α- and β-tubulin heterodimers (16, 17). Microtubules extend from the microtubule-organizing center located in the centrosome and interact with various organelles including endoplasmic reticulum, Golgi apparatus, lysosomes, and mitochondria (18–20).

The main feature of microtubules is their dynamics, they constantly shrink and expand by reversible connection and disconnection of α- and β-tubulin heterodimers (21). The unique structure of microtubules makes the dynamics of tubulin heterodimer release and addition slower at the (−) end and quicker at the (+) end (22, 23). The shrinking phase of the microtubule is called “catastrophe” and is defined as a transition from lengthening to shrinking period at the (−) end of the microtubule (24). Conversion from shrinking to lengthening period at the (+) end is known as the growth phase, or “rescue.” The microtubule dies if it does not undergo a transition between these two states.

The disassembly of microtubules is accompanied by the formation of the new network of spindle microtubules that are much more dynamic than interphase microtubules (as high as 100 times)!. This process leads to the creation of mitotic spindles. Production of mitotic aster and centromeric microtubules requires stringent regulation of microtubule dynamics to assure individual chromosome attachment and segregation during cell division (25). In addition, the natural dynamics of microtubule fibers permit conventional segregation of chromosomes. Failure to accurately attach or separate chromosomes initiates the arrest of the cell cycle in the mitotic checkpoint, resulting in apoptosis (26, 27).

There is a multitude of different regulatory proteins that play an important role in microtubule structural stability: promoting microtubule stability proteins, such as (−) terminal combined with gamma-tubulin and gamma-tubulin compound protein (GCPs), a lateral combination of microtubule-associated protein 2 (MAP2) and τ protein; (+) terminal-binding stabilizing microtubule proteins, such as beta TIPs (EB1 and CLIP170), etc. There are also microtubule-binding polymerizing or depolymerizing proteins, such as cleaved enzymes (spastin and katanin), (+) terminal depolymerizing kinesin-13, and α/β-microtubule dimer stabilizing protein stathmin (28, 29). Microtubules interact with the proteins involved in intracellular transport (kinesins and dyneins), cell cycle, and apoptosis regulatory proteins, including tumor suppressor protein p53, which connects directly to dynein and also interacts with Bcl-2, survivin, and other prosurvival proteins (30). However, the nature and function of these interactions between dividing cells and tumor cells are not clear and deserve further investigation (31, 32).

## Isotypes of Tubulin and Their Functions

In humans, microtubules are composed of various tubulin isoforms:α-tubulin, β-tubulin, γ-tubulin, δ-, ϵ-, and z- tubulin (33). The heterodimers of α- and β-tubulin are the basic structural components that constitute microtubules and control their functions. The members of the tubulin family differ from one another by sequences at the C-terminal tail that functions as a binding domain for microtubule-associated proteins (MAPs) (34).

The composition of tubulin dimers and microtubules, their dynamics, and functions are affected by the expression of tubulin isotypes. Tubulin isotypes can undergo detyrosination, glutamylation, glycylation, acetylation, and other kinds of post-translational modifications that can affect MAPs (35). Multiple studies established that altered expression of certain tubulin isomers and MAPs are associated with cancer (36–38). As a result, altered expression of different tubulin isotypes is implicated with drug resistance. However, the exact mechanism of developing isotype-specific resistance is still not clearly understood, uncovering it is a key to creating novel cancer biomarkers and drugs.

### α-Tubulin

The function of α-tubulin isotypes and their role in cancer require further investigation. Only several studies researched the expression of α-tubulin isotypes in cancer or normal tissues. The expression levels of α-tubulin isotypes are associated with sensitivity to anti-tubulin agents and poor prognosis in many types of cancer. For instance, some studied found a correlation between the high expression level of α1B-Tubulin and the poor prognosis in hepatocellular carcinoma and mantle cell lymphoma (36, 39). Upregulated expression of α1C-tubulin predicts poor prognosis and promotes proliferation and migration in hepatocellular carcinoma (40). Expression of α3C-tubulin is associated with the decreased response of ovarian cancer to paclitaxel (41). High expression of kα-1-tubulin affected paclitaxel therapy for anaplastic carcinomas. Finally, Δ2α-tubulin level is related to poor response to drugs binding to the vinca alkaloid site in the treatment of advanced non-small-cell lung carcinoma (NSCLC) (42).

### β-Tubulin

β-tubulin isotypes have been studied more comprehensively than α-tubulins. Increased expression of β-tubulin isotypes was found in different tumors. Specimen analysis and clinical research determined that high production of β-tubulin isotypes, such as βI-, βII-, βIII-, βIVa-, and βV-tubulin, are associated with disease progression, aggressive clinical behavior, overall survival, poor patient outcome, and chemotherapy resistance. Recent studies concluded that tumor aggressiveness, uncontrolled cell proliferation, and malignant biological behaviors of tumor cells, such as infinite growth, invasion, metastasis, and resistance to chemotherapeutic agents, are closely correlated with abnormal expression and distribution of β-tubulin isotypes. βIII-Tubulin (TUBB3) is the most commonly found highly expressed β-tubulin isotype that is related to cancer. The altered expression level of TUBB3 was observed in many human cancer cells, and its aberrant expression was found to be associated with enhanced chemoresistance and poor prognosis in NSCLC, ovarian cancer, gastric cancer, breast cancer, and uterine serous carcinoma (43–46). Moreover, increased expression of TUBB3 is associated with glioblastoma, colorectal cancer, and PDAC (47, 48). High expression of βII-Tubulin (TUBB2) was shown to be correlated with decreased overall survival in colorectal cancer (49). Several studies established a strong association between decreased TUBB2 expression and advanced stage of ovarian cancer, as well as resistance to taxane treatment in ovarian cancer (50, 51). Breast cancer cells were shown to have decreased response to docetaxel treatment in patients with high βI-tubulin expression (46). Furthermore, overexpression of βIVa-Tubulin (TUBB4) is correlated with the poor response of paclitaxel treatment in patients diagnosed with ovarian cancer and NSCLC (52, 53).

## Tubulin in Pancreatic Cancer

Recent studies have determined the roles of TUBB2, TUBB3, and TUBB4 in PDAC. Immunohistochemical studies showed that these β-tubulin isotypes are more highly expressed in PC tissues than in paracancerous tissues. Also, they are upregulated in PC cell lines and downregulated in normal human pancreatic duct epithelial (HPDE) cell lines (37, 38).

### βIII-Tubulin

The Western blot and RT-PCT showed different expression levels of TUBB3 in PC and HPDE cell lines. TUBB3 was upregulated in PC cells lines and downregulated in the latter. It was demonstrated that the knockdown of TUBB3 decreased the growth of cell colonies. The number of colonies significantly decreased following the administration of chemotherapy drugs (gemcitabine, paclitaxel) (47). Knockdown of TUBB3 expression in PC cells leads to anchorage-independent and -dependent cell growth related to enhanced anoikis (anchorage-independent apoptosis), thus strengthening the link between suppressed TUBB3 and initiation of apoptosis in PC cells. TUBB3 shRNA decreased tumorigenic potential, tumor growth, and metastases of PC cells in a xenograft mouse model (37).

### βIV-Tubulin

βIV-Tubulin isotype includes two subtypes: tubulin βIVa (TUBB4) and βIVb-tubulin (TUBB2C). βIV-tubulin is highly expressed in all PDAC cell lines (MiaPaCa-2, HPAF-II, and AsPC1) compared with HPDE ones. TUBB2C plays an important role in regulating PC cells’ anchorage-dependent growth and responsiveness to chemotherapeutic drugs (37).

Notably, knockdown of TUBB2C largely influences PDAC cell growth and chemosensitivity. In particular, the knockdown of TUBB2C can enhance the sensitivity of HPAF-II and AsPC1 cell lines to paclitaxel and gemcitabine, while no effect was observed in MiaPaCa-2 cell lines. Also, TUBB2C may play a role in modulating chemoresistance in certain subtypes of PDAC cell lines. For example, knockdown of TUBB2C sensitizes all PDAC cell lines to vincristine by initiating apoptosis in tumor cells. Further examination showed elevated sensitivity to other vinca alkaloids, including vinorelbine and vinblastine. Knockdown of TUBB2C does not affect normal pancreatic HPDE cell proliferation. In other words, the drug’s anti-proliferative properties are highly specific to cancer cells. Hence, knockdown of TUBB2C can induce the ability of the vinca alkaloids to arrest mitosis and induce apoptosis. In summary, these results contribute to opening new possibilities for PDAC therapy. TUBB2C is likely to become an object of thorough research of therapeutic targets that may increase the sensitivity of PDAC cells to ligands binding to the vinca alkaloid site (38).

### Therapeutic Efficiency of a Novel βIII/βIV-Tubulin Inhibitor (VERU-111)

Just like in other cancers, tubulins play a significant role in the progression of PDAC. Among all the tubulins, βIII and βIV isotypes may have the strongest association with PDAC progression, metastasis, and chemoresistance (54, 55). Therefore, the ability to selectively target βIII and βIV tubulins may improve the therapeutic response of PDAC. Recently, a novel βIII and βIV inhibitor, VERU-111, was created. VERU-111 can efficiently suppress the growth of aggressive PC cells. qPCR and Western blot analysis demonstrated potent inhibitory properties of VERU-111, which arise from its ability to affect the expression of all the β-tubulin isotypes (56). Another study found that miR-200c was significantly restored in PDAC cells after VERU-111 administration (p < 0.01), whereas miR-200c inhibitor could decrease the effect of VERU-111 on the expression of TUBB3. This indicates that VERU-111 most likely targets TUBB3 *via* miR-200c (57).

VERU-111 causes cell cycle arrest in the G2/M phase, which is somewhat similar to other microtubule-targeting agents (MTAs). Due to arrest in the G2 phase, cancer cells can no longer repair DNA damage, so they move directly into the M phase, making the G2/M checkpoint a suitable target for anti-cancer drugs.

VERU-111 also affects the expression of Cdc2, cyclin B1, and Cdc25C kinases (58). Flow cytometry data showed that VERU-111 induces apoptosis in PDAC cells *via* altering mitochondrial proteins (Bcl-xL, Bcl-2, Bax, and Bad). Additionally, it can activate caspase-3, caspase-9, and cleavage of PARP that are essential in the apoptotic pathway (59). These outcomes suggest the involvement of multiple apoptosis-related proteins in the death of PDAC cells caused by VERU-111.

Xenograft mouse model results showed that VERU-111 (50 μg/mice) can effectively suppress tumor growth along with suppression of βI, βIII, and βIV tubulins and restoration of miR-200c expression. Taken together, VERU-111 suppresses pancreatic tumor growth *via* influencing cell cycle arrest, restoring miR-200c, and inducing apoptosis of PDAC cells, which may be efficacious in PDAC treatment (56).

## Microtubules as Targets in Cancer Chemotherapy

**Figure 1:**
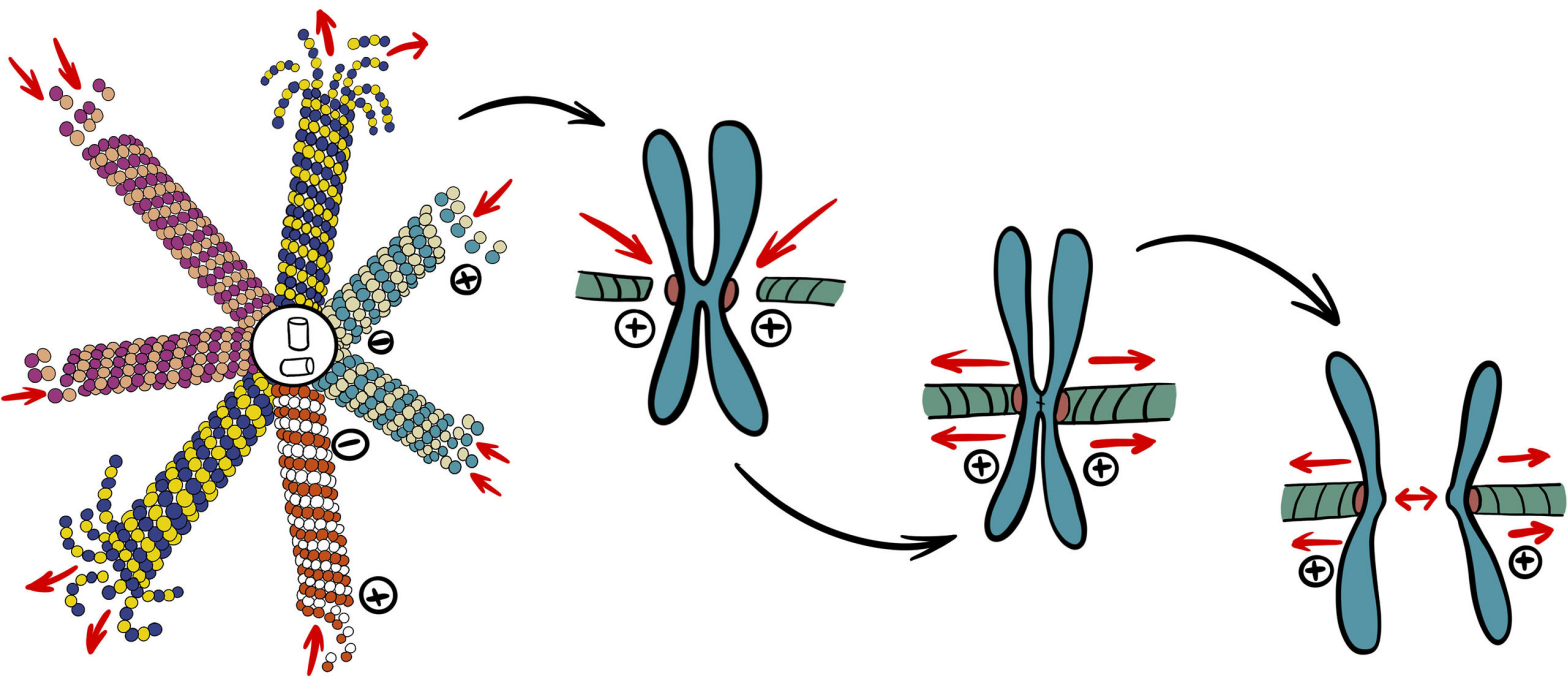
Simplified role of microtubules in mitosis. Catastrophe rate of cytoplasmic microtubules increases to provide building blocks to different populations of spindle microtubules required for mitosis. Nuclear envelope breakdown allows spindle microtubules to attach to kinetochores of chromosomes. After chromosomes are aligned at equator, chromatids can finally segregate through depolymerization of attached microtubules and spindle pole movement.

Microtubules have become one of the core approaches in cancer pharmacology and targeted therapy due to their pivotal role in mitotic cell division (60). As the cell undergoes prophase, microtubules existing in the cytoplasm begin to depolymerize more rapidly (61). This highly dynamic process is crucial for the assembly of the mitotic spindle, prompt and complete segregation of chromosomes during cell division. In the following stage of division, spindle microtubules pull the sister chromatids from the equator to the two poles of the spindle (**Figure 1**). The end of mitosis is marked by depolymerization of spindle microtubules as they assemble back into cytoplasmic microtubules. The dynamic characteristics of depolymerization and polymerization are necessary for cells to complete mitosis (62).

If this cycle is interrupted, the cell will not enter mitosis, or cell division will be disrupted followed by mitotic arrest or division errors, decreased proliferation, and cell death (60). Impairment in the dynamic behavior of microtubules affects the division of tumor cells and inhibits their growth. Therefore, microtubules are believed to be one of the most promising targets in cancer. Most of the anti-angiogenic agents in clinical trials are MTAs.

Microtubule inhibitors comprise a highly effective class of anti-cancer drugs and have been widely applied in the treatment of hematopoietic and solid tumors. The majority of these MTAs are anti-mitotic agents that induce cell cycle arrest in the G2/M phase and produce irregular mitotic spindles (63). They disrupt the structure of microtubules and inhibit cell proliferation by alternating polymerization dynamics of spindle microtubules (54). Most MTAs can be classified into two groups: microtubule-destabilizing agents (MDAs) and microtubule-stabilizing agents (MSAs) (**Table 1**).

**Table 1:** Microtubule-targeting agents in pancreatic cancer.

	Name	Origin	Anti-cancer properties	Clinical trials
**Taxane site**	Paclitaxel	*Taxus brevifolia*	Probably p53 stimulation	Approved by FDA for pancreatic cancer
	Nab-paclitaxel	Paclitaxel (*Taxus brevifolia*)	Probably p53 stimulation	Nab-paclitaxel + gemcitabine is approved by FDA for late-stage pancreatic cancer
	Epothilones	*Sorangium cellulose*	Apoptosis induction (probably Bcl-2 targeting)	Ixabepilone completed phase II clinical trial (64)
	10ae	Synthetic	Anti-proliferative, apoptosis induction (caspase family activation)	Pre-clinical
**Colchicine site**	NSC 51046	Colchicine (*Gloriosa superba*/*Colchicum autumnale*)	Apoptosis induction, anti-vascular	Pre-clinical. Earlier, phase II of structurally similar ZD 6126 was suspended (65)
	UA62784	Synthetic	Anti-proliferative, apoptosis induction	Pre-clinical
	Plinabulin	Synthetic (*Aspergillus ustus*)	Anti-proliferative	Phase II and III clinical trials against non-small-cell lung carcinoma
	TH-482, TH-337, TH-494	Synthetic (indazole)	Anti-proliferative, anti-vascular	Pre-clinical
**Vinca alkaloid site**	DZ-2384	AB-5 (synthetic (*Diazonaangulata*))	Anti-proliferative	Pre-clinical

## Microtubule-Destabilizing Agents in Pancreatic Cancer

Compounds that inhibit microtubule polymerization and reduce microtubule polymer bulk are known as MDAs. Agents in this group arrest the formation of mitotic spindles by acting on different binding sites of microtubules, mainly colchicine and vinca alkaloid sites (66). Two additional binding sites exist (pironetin site and maytansine site), but there are no known agents active against PC that can bind to them.

### Colchicine Site

**Figure 2:**
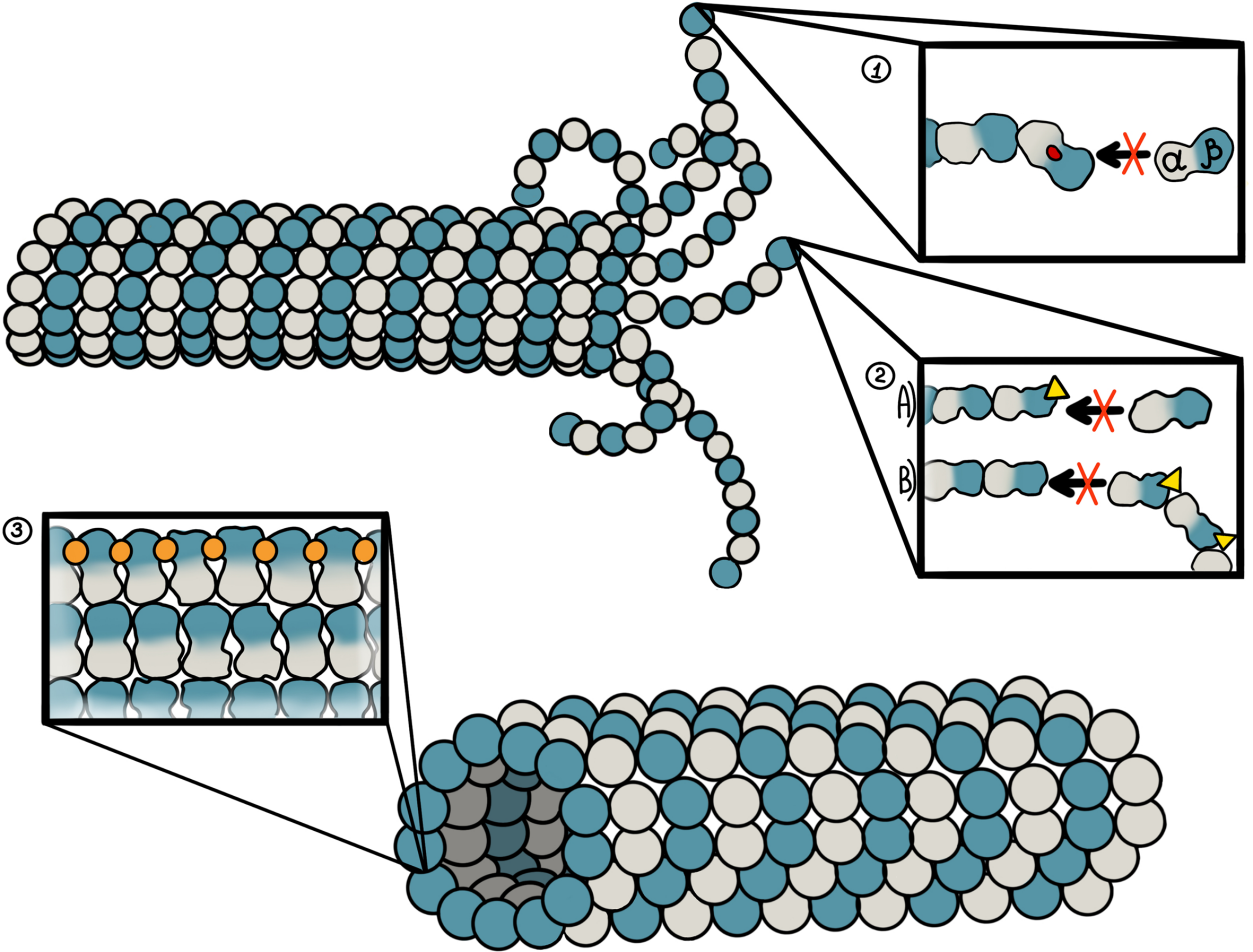
Binding sites of microtubule-targeting agents against pancreatic cancer and their mechanism of action. 1. Colchicine site. It is located at the junction of the α-β subunit of the microtubule, which is adjacent to the GTP binding site on the α-subunit. Colchicine-site ligands inhibit microtubule polymerization by preventing “curved-to-straight” transition. 2. Vinca alkaloid site. The binding site of vinca alkaloids is located near the GTP binding site of β-tubulin. Vinca alkaloids display two mechanisms of action. First, binding of vinca alkaloid ligands introduces a wedge at the end of microtubules, thus preventing a “curved-to-straight” transition. Second, the binding of vinca alkaloids results in ring-like tubulin oligomers that cannot assembly into the microtubule. 3. Taxane site. This site is located at the β-tubulin pocket facing the lumen of microtubules. Taxoids stabilize M-loop and thus promote microtubule assembly.

The colchicine site is occupied by the majority of MDAs. It is located at the junction of the α-β subunit of the microtubule (**Figure 2**) (67). Binding to the β-tubulin results in the original straight conformation bending, causing steric hindrance between colchicine and α-tubulin. This binding first occurs on the α/β-tubulin dimer, which does not participate in the formation of the microtubules (68). The stable complex formed at the end of microtubules significantly reduces the microtubule capacity to polymerize. Notably, colchicine can also induce microtubule depolymerization by inhibiting the interaction between the microtubule fibrils (69). Some of the most well-known drugs that bind to this site are colchicine, 2methoxyestradiol (2ME), combretastatin-A4 (CA-4), combretastatin-A2 (CA-2), and podophyllotoxin (PDT).

### N-Acetyl-O-Methylcolchinol

Derivatives of colchicine have potent activity in many types of cancer cells, including PDAC cell lines. N-acetyl-O-methylcolchinol (NSC 51046) was reported to block mitosis by inhibiting microtubule polymerization. It can hinder the cell cycle of PC cells and trigger apoptosis. NSC 51046 inhibits tubulin polymerization at low doses and, strikingly, also promotes tubulin polymerization at higher doses. NSC 51046 induces apoptotic cell death in approximately 70% of both PC cell lines (PANC-1) and normal fibroblasts. Nonetheless, NSC 51046 displays non-selective properties and mild potent activity, preventing it from becoming a part of targeted therapy. Its analogs might prove different (65).

### UA62784

UA62784 is one of few compounds that selectively target PDAC cells. It is a novel highly potent microtubule inhibitor with enormous cytotoxicity whether used alone or with other MDAs. Its cytotoxicity is additive to that of vinca alkaloids and may solve the problem of cancer cells’ resistance to MDAs.

Intrinsic tubulin tryptophan fluorescence experiments demonstrated the ability of UA62784 to bind to α- and β-tubulin dimers. UA62784 similarly to other MDA compounds, such as vinblastine, nocodazole, and colchicine, induced a fluorescence quenching upon binding to the α- and β-tubulin dimmers. UA62784 displayed a high affinity of 27 ± 13 nM for tubulin in a model with a high-affinity site with a dissociation constant in the nanomolar range (Kd1) and of 142 ± 104 μMin a model with a low-affinity site with a dissociation constant in the micromolar range (Kd2). Since Kd2 value is greater than Kd1 by more than 5,000 times, the former could be left out for UA62784. Kd1 values for known microtubules anti-tumor agents, such as colchicine (324 ± 36 nM), vinblastine (227 ± 45 nM), and nocodazole (259 ± 86 nM), show an approximately 10 times lower affinity compared with UA62784. On the other hand, the affinity of the second binding mode (Kd2) is two-fold (colchicine and vinblastine) to eight-fold (nocodazole) higher than UA62784, suggesting the possibility of the existence of the second binding site.

[3H]-colchicine experiment showed that the synergy of [3H]-colchicine with tubulin is reduced by the addition of raising doses of UA62784. The addition of 2 to 4 μM of UA62784 compound dismisses more than 60% of the tubulin heterodimers bound to [3H]-colchicine, which is similar to the results seen after administration of 10 μM of colchicine. On the contrary, even high doses of vinblastine do not alter the interaction of [3H]-colchicine with tubulin. This confirms that UA62784 directly combines with tubulin heterodimers at the colchicine-binding site and affects the number of microtubules *in vitro*.

Flow cytometry assay revealed that 20 nM of UA62784 for 12 h increases the doubling in the G2/M phase from 21.5% ± 2.8% to 40.1% ± 1.1% in untreated HeLa cells. At a higher dose of 200 nM for 24 h, UA62784 promotes the accumulation of phosphorylated histone H3, cyclin B, and MPM2. The presence of another mitotic marker, phosphorylated BubR1/BUB1B, strongly indicates that UA62784-treated tumor cells undergo mitotic arrest due to activated spindle assembly checkpoint (SAC). Moreover, β-tubulin staining showed that the administration of UA62784 promotes microtubule depolymerization in PC cells (Panc-1 cell lines). Finally, a relatively simple structure of UA62784 makes it an appealing agent in terms of structural modifications and modeling (70, 71).

### Plinabulin

Plinabulin is isolated from a fungal metabolite from *Aspergillus ustus*. Recently, a combination treatment consisting of docetaxel and plinabulin has entered phase III trial for NSCLC therapy. Plinabulin has to be administered by intravenous injection due to its poor water solubility. Synthetic derivatives of plinabulin, compounds 1 and 2, display activity against cancer cells (inhibition percentage in human BxPC-3 PC cell lines – >85% at 12.5 nM). Compound 1 at the IC50 value of 0.63 nM exhibited stronger anti-tumor activity than plinabulin at that of 4.28 nM (72, 73).

### TH-482, TH-337, and TH-494

TH-482, TH-337, and TH-494 are lead compounds that belong to indazole-based microtubule inhibitors. They have potent anti-proliferative activity against PC cells (MIA PaCa-2 cell lines). TH-482 has the most potent anti-proliferative activity in 11 cell lines, including MIA PaCa-2 cell lines. It was shown to inhibit tubulin polymerization *in vitro* and lead to arrest in the G2/M phase. In addition to its effect on the cell cycle, TH-482 exhibits vascular-disrupting activity *in vitro*. It hinders angiogenesis, increases endothelial cell permeability, and destroys pre-existing vasculature. Remarkably, all of this can be achieved only with nanomolar TH-482 concentrations. At the same time, micromolar concentrations of TH-482 are required for the inhibition of microtubule polymerization. These findings are no different from other MTAs, such as paclitaxel, epothilones, combretastatin-A4 (CA-4) sulfonate analogs, T138067, and 2-(3,4,5-trimethoxybenzoyl)-3-amino 5-aryl thiophenes (74).

### Vinca Alkaloid Site

Experimental studies determined that vinca alkaloids mainly bind to amino acid residues at the 175-213 position of β-tubulin. They induce microtubules depolymerization at high concentrations (75). Major drugs of this group are vinblastine, vindesine, vinorelbine, vinflunine, and vincristine. Eribulin mesylate (Halaven^®^), which acts on the vinca alkaloid site, was approved by the FDA in 2010 for the treatment of breast cancer.

### DZ-2384

DZ-2384 is a synthetic derivative of AB-5. AB-5 has long been known to have anti-tumor activity in animal xenograft models. Its precursor is diazonamide A, another sponge-isolated compound. DZ-2384 synthesis is simplified and commercially scalable compared with earlier diazonamide analogs (76). Unlike vinorelbine, it can increase the rescue rate, and the produced difference in microtubule dynamics is greater than in other MDAs (dolastatin 10, vincristine, etc.).

DZ-2384 has anti-tumor activity in PC xenograft models and other types of cancer in various models such as a patient-derived xenograft model and a genetically engineered mouse model with immunocompetent mice. DZ-2384 binds to the vinca alkaloid site in a unique way, producing higher anti-tumor properties and safety. The electron microscopy and X-ray crystallography showed that DZ-2384 modifies the curvature between tubulin dimers, thus straightening protofilaments. It enhances the rescue frequency, and, despite the limited effect on microtubules destabilization compared with vinorelbine, it is adequately sufficient to disrupt mitotic spindle formation. As a single agent alone, DZ-2384 has anti-tumor activity in MIA PaCa-2 cell lines. Researchers reported that DZ-2384 induced complete neoplasm regression in the MIA-PaCa-2 xenograft model, and all the mice were cancer-free ~3 months after therapy (9 mg/m^2^). Vinorelbine was also effective in both xenograft models, but at higher doses and for a shorter term. The importance of DZ-2384 also lies in its increased safety margin (more than 24-fold vs 0.7- to 1.0-fold in vinorelbine) in terms of weight loss, prognosis, bone marrow toxicity, and more than 13-fold in terms of neurotoxicity. A combination of DZ-2384 and gemcitabine was observed to be more efficacious than gemcitabine monotherapy, which is the first-line treatment of patients with PDAC. DZ-2384 together with gemcitabine decreased tumor formation and progression with a higher response rate (68%) than a combination of nab-paclitaxel and gemcitabine (53%) in Rgs16::GFP; KIC model. These results are indicative of DZ-2384 being a possible candidate for PDAC treatment and its potential to be used in a wide range of other applications (77).

## Microtubule-Stabilizing Agents in Pancreatic Cancer

### Taxane Site

MSAs influence cell proliferation by inhibiting cell division and blocking the cell cycle in the G2/M phase, producing abnormal mitotic spindle afterward and leading to cancer cell death *via* apoptosis. MSAs mainly promote the polymerization of microtubules, making them unusually stable and increasing their quantities in the cell (78). So far, only the taxane-site ligands were shown to have potent activity against PDAC.

### Paclitaxel

The representative drug of the taxane drugs is paclitaxel (Taxol^®^). The structure of paclitaxel was discovered in 1971, but its microtubule-stabilizing characteristics were identified only 8 years later, in 1979 (79). It easily binds to the assembled microtubules on the β-tubulin subunit. Generally, the process of microtubule polymerization requires GTP, but paclitaxel can promote tubulin polymerization without it. Paclitaxel promotes microtubule polymerization at low concentration and temperature without significantly rising polymer levels of the microtubule (78, 80).

Paclitaxel is one of the most effective microtubule-targeting anti-cancer drugs. Paclitaxel was approved by the FDA in 1992 and is stillconsidered to be one of the most critical supplements to chemotherapeutic regimens against various cancers, including PC (81). At present, paclitaxel combined with albumin-based chemotherapy is used as the first line of advanced PC therapy. Paclitaxel influences the dynamics and microtubule polymerization *via* binding to the taxane site, which leads to cell cycle arrest and cell death. Because paclitaxel dramatically decreases cell proliferation and mitotic rate of microtubules at low concentrations without significantly rising polymer levels, suppression of microtubule dynamics appears to be its most effective mechanism of mitotic arrest. Paclitaxel at high concentrations promotes the addition of tubulin dimers and disturbances ina dynamic balance of microtubules but acts the opposite at low concentrations (82). Several approaches have been implemented to improve the solubility and pharmacology of paclitaxel, including albumin nanoparticles, liposomes, and emulsions (81). Albumin-stabilized nanoparticle formulation of paclitaxel is also known as ABI 007, or nab-paclitaxel.

Tumors harvested from untreated animals group were stained with Collagen IV and Masson’s trichrome. Researchers found enormous levels of fibrotic tissue in the tumor microenvironment. The visually impressive decrease in fibrotic tissue mass was noted in tumor tissues after administration of nab-paclitaxel compared with those treated withpaclitaxel. Nab-paclitaxel therapy decreased the amount of proliferating carcinoma cells to a greater extent than paclitaxel therapy as evidenced by a decreased amount of carcinoma cells expressing Ki-67. Nab-paclitaxel plus gemcitabine therapy was very effective in inhibiting Ki-67 (+) tumor cells compared with paclitaxel plus gemcitabine treatment. Plasma and intratumor concentrations of paclitaxel following nab-paclitaxel or paclitaxel therapy were performed to investigate the potential mechanism of the therapeutic effectiveness of nab-paclitaxel over paclitaxel. Nab-paclitaxel therapy was correlated with higher tumor stroma in the tumor microenvironment compared with paclitaxel-treated and untreated tumors.

According to the results from both clinical and preclinical studies, the efficacy of nab-paclitaxel is superior to that of cremophor-based paclitaxelowing to many factors including a better pharmacokinetics behavior. A higher intratumor paclitaxel concentration was achieved after nab-paclitaxel treatment that resulted in desmoplastic tumor stroma destruction and enhanced neoplastic cell death. This may be another reason for the superiority of nab-paclitaxel over paclitaxel treatment in PDAC (81).

### Nab-paclitaxel

Nab-paclitaxel (Abraxane^®^) is a 130-nm, solvent-free, albumin-bound formulation of paclitaxel. Apart from hindering cell division *via* interrupting the microtubule network, it can enhance transportation of paclitaxel to endothelial and tumor cells. Nab-paclitaxel has many advantages compared with sb-paclitaxel. For instance, it produces significantly higher doses of paclitaxel in a shorter transfusion time (30 min vs 3 h for sb-paclitaxel), it can reach a higher peak concentration, enhance drug combination to tumors and endothelial cells more effectively. Another study showed the nab-paclitaxel has a higher neoplasm uptake thansb-paclitaxel after administration at equal doses.

In phase I and II trials, a maximum-tolerated dose of nab-paclitaxel and gemcitabine (1000 and 125 mg/m^2^, respectively) was given to advanced PDAC patients (QW 3/4 w). 44 patients had an overall response rate of 48% and a median overall survival of 12.2 months. In phase III study, 850 patients with metastatic PDAC receiving the same regimen were compared with monotherapy of gemcitabine 1000 mg/m^2^ (QW 7/8 was cycle 1 and QW3/4 was cycle 2). Median overall survival was significantly longer in the nab-paclitaxel plus gemcitabine group (8.5 vs 6.7 months). Other trials reported that the grade 3 neuropathy was correlated with nab-paclitaxel treatment in a majority of patients with advanced PC (83).

Effects of gemcitabine and nab-paclitaxel were investigated in the following PDAC cell-lines: MIA PaCa-2, AsPC-1, BxPC-3, and Panc-1a. Addition of nab-paclitaxel or docetaxel at IC25 reduced IC50 of gemcitabine. Tumor growth inhibition after gemcitabine, nab-paclitaxel, and docetaxel was 67%, 72%, and 31%, respectively. Tumor stromal mass (estimated through the reduction in α-smooth muscle actin, collagen I, and S100A4 expression) was reduced more greatly by nab-paclitaxel than docetaxel. Furthermore, a PDAC xenograft model study showed that nab-paclitaxel is more efficacious and results in longer median survival than gemcitabine. Phase I, II, and III trials were performed to examine nab-paclitaxel-based chemotherapy together with target therapy or immunotherapy in metastatic PDAC patients (84). Nab-paclitaxel plus gemcitabine therapy comprises standards of metastatic PC care, and this combination is suitable for PDAC patients with different characteristics and clinical presentations (85).

Secreted protein acidic and rich in cysteine (SPARC) has a crucial role in the transport of nab-paclitaxel to a tumor. A research was conducted to examine the relationship between the prognosis of patients receiving nab-paclitaxel plus gemcitabine and SPARC expression (83). In phase I and II, stromal SPARC expression (high and low) was significantly associated with OS in the nab-paclitaxel plus gemcitabine group (17.8 vs 8.1 months), indicating that SPARC may serve as a biomarker for PC. However, phase III concluded that intratumor, stromal, and plasma SPARC were not predictive of survival rate in both groups with metastatic PC.

As technologies advance, nab-paclitaxel undergoes additional investigations for PDAC therapy. The solvent-free albumin-paclitaxel nanoparticles are comparatively more favorable than solvent-based formulations of cre-paclitaxel in patients with advanced metastatic PC. Stopping treatment with albumin-paclitaxel is associated with a lower risk of neutropenia, infusion hypersensitivity responses, and quicker alleviation of external neuropathy. Albumin-paclitaxel is currently regarded as an ideal regimen for patients with metastatic PDAC. Albumin-bound formulation reduces tumor stroma *via* synergy between albumin and SPARC, thereby affecting the tumor microenvironment. This mechanism promotes the gemcitabine-enhanced effect.

Several studies examined the efficacy and survival advantage of nab-paclitaxel alone and in combination with gemcitabine. They aimed to study treatment effects on tumor cell proliferation, tumor desmoplasia, and metastases to adjacent organs (86). Nab-paclitaxel as an individual agent was not found to be significantly useful in decreasing primary tumor weight or increasing mouse survival rate compared with nab-paclitaxel or gemcitabine monotherapy. Finally, combined treatment of gemcitabine and nab-paclitaxel reduced metastatic tumor burden and elevated median survival rate of animals greater than any of the agents alone (87, 88). The synergy between nab-paclitaxel and gemcitabine in PDAC was assessed in two preclinical models: genetically engineered mice and primary patient-derived tumors. The result of the experiment in a primary tumor xenograft model demonstrated that nab-paclitaxel plus gemcitabine induced regression of the tumor in 64% of the 11 biologically different primary tumors versus 36% and 18% nab-paclitaxel and gemcitabine monotherapy. Another study’s outcomes showed that nab-paclitaxel treatment was more effective in preventing initial tumor progression, solid tumor stroma depletion, consistently showing a higher anti-tumor response and increased the survival rate in animal models than paclitaxel treatment. Combined treatment of gemcitabine plus nab-paclitaxel reduced metastatic tumor burden and improved the overall survival rate of animals compared with monotherapy of any of the agents. Moreover, there is no benefit of adding paclitaxel to gemcitabine treatment for regionally advanced and metastatic PDAC (82). In 2013, gemcitabine plus nab-paclitaxel was approved by the FDA as the first-line treatment for patients with metastatic PC. Nab-paclitaxel plus gemcitabine could better improve tumor response and survival rates in metastatic PDAC than gemcitabine alone (89).

Treatment with nab-paclitaxel seemed to exhaust the desmoplastic stromal matrix and improve microvasculature in gemcitabine-resistant primary tumors. Intratumoral gemcitabine concentration was 2.8 times higher in mice receiving nab-paclitaxel plus gemcitabine than gemcitabine alone. Related synergistic anti-tumor and pharmacologic responses were confirmed in a transgenic PDAC murine model. The results showed that paclitaxel elevated intratumoral accumulation of gemcitabine *via* inactivation of cytidine deaminase. Another study revealed that nab-paclitaxel plus gemcitabine therapy efficiently reduced the density of tumor-associated fibroblasts and produced disruptive changes in tumor stroma. Preclinical trial results revealed the positive anti-tumor activity of nab-paclitaxel and its potential to alter desmoplastic stroma. This was part of the MPACT trial, a randomized phase III study, which confirmed the efficacy of nab-paclitaxel plus gemcitabine. The results of this research lead to regulatory approval of nab-paclitaxel plus gemcitabine therapy, which is now a standard regimen for metastatic PC (87).

### Epothilones

Epothilones are a novel class of anti-microtubule agents derived from the soil bacterium *Sorangium cellulose*. They bind to the taxane site and stabilize microtubule polymerization. Epothilones have the activity of promoting assembly and polymerization of microtubules. After binding to the microtubule, epothilones restructure the disordered M-loop (site of lateral tubulin contacts) within the microtubule and stabilize it. Compared with paclitaxel, epothilones have several advantages. First, the activity of epothilones is 10 to 1,000 times higher than that of paclitaxel. Second, the water solubility is also higher. Lastly, the structure is much simpler, making them easier to synthesize (90). Hence, epothilones were introduced to the Oncologist’s portfolio of drugs, which represents a pivotal step in PDAC therapy.

A human PC xenograft model experiment demonstrated the effectiveness of one of the derivatives of epothilone B against PDAC. This derivative is named ixabepilone, previously known as BMS-247550. Ixabepilone is more efficient in inhibiting tumor growth than paclitaxel with five to six lower doses required in mice and rats. Phase II trial (Southwest Oncology Group) suggested that ixabepilone is efficacious in the treatment of patients with PC. The treatment with this compound resulted in median survival of 7.2 months and 6-month survival of 60%, whereas the patients receiving gemcitabine had a median survival of 5.65 months and 6-month survival of 46% (91).

### (Z)-1-(2-bromo-3,4,5-trimethoxyphenyl)-3-(3-hydroxy-4-methoxyphenylamino)-prop-2-en-1-one (10ae)

(Z)-1-aryl-3-arylamino-2-propen-1-one (10) compounds enhance microtubule stability and induce cell apoptosis *via* caspase family (92). (Z)-1-(2-bromo-3,4,5-trimethoxyphenyl)-3-(3-hydroxy-4-methoxyphenino)-prop-2-en-1-one (10ae) promotes tubulin polymerization and induces apoptotic cell death in MIA-Paca2 and Panc-1 cell lines (93).

10ae has a remarkable cell killing ability. It induces apoptosis in 20 tumor cell lines with similar GI50 values, including drug-resistant tumor cell lines. Such a broad spectrum of action is probably due to its inhibitory effect on critical stages of cancer cell division. Flow cytometry demonstrated that 10ae arrests cells in the G2/M phase and that it may trigger apoptosis through activation of caspases (50).

Treatment with 10ae leads to tumor cell accumulation in the G2/M phase in a dose-dependent manner. Tumor cells begin to accumulate in the G2/M phase shortly after administration of 0.25 μM of 10ae, whereas G2/M arrest occurs after treatment with twice the dose. The fluorescence-activated cell sorting showed that treated tumor cells had >2N DNA regardless of whether it was G2 or M phase. The influence of 10ae on the phosphorylation of proteins serving as markers of SAC activation and mitotic arrest (history H3, Bcl-2, and BubR1) was then studied. All three were phosphorylated 6 h after 10ae treatment, showing a similar increase in concentration level (2.95-fold). To conclude it all, 10ae has the most potent cytotoxic properties among the other derivatives of 10 compounds. It also surpasses paclitaxel in that it leads to greater tubulin polymerization at the same concentration (1 μM) (92).

## Mechanism of Microtubule-Targeting Drugs in Cancer Therapy

### Block Cell Mitosis and Cycle Progression

**Figure 3:**
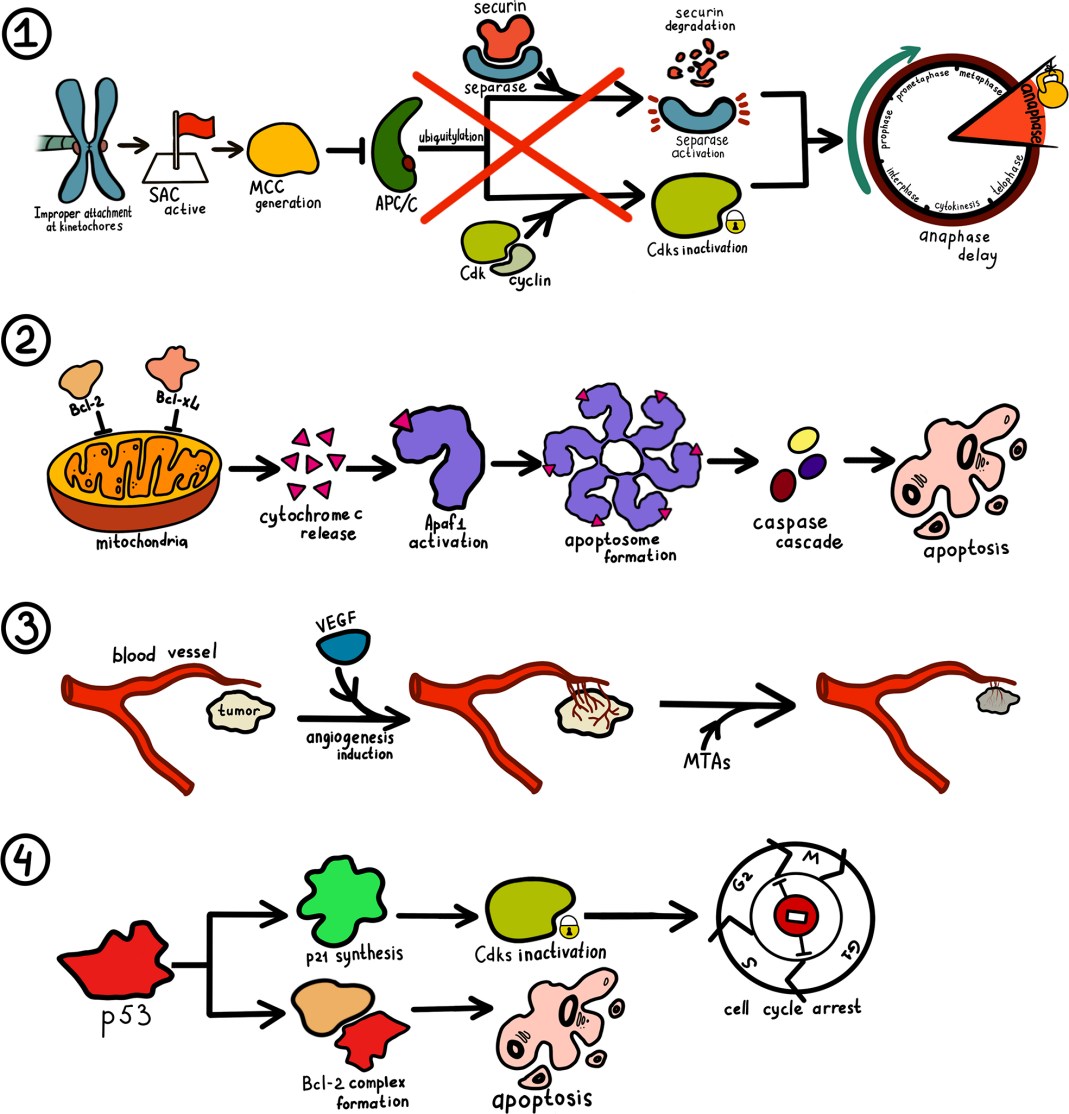
Mechanism of microtubule-targeting drugs in cancer therapy. 1. Improper, incomplete or absent attachment at kinetochores maintain spindle assembly checkpoint (SAC) activity. When SAC is active, a group of checkpoint proteins constituting mitotic checkpoint complex (MCC) is recruited and block the activity of anaphase-promoting complex/cyclosome (APC/C). As ubiquitylation of cyclins and securin does not take place, a cell’s entry into anaphase is impossible. 2. MTAs trigger phosphorylation of Bcl-2 and Bcl-xL, allowing for cytochrome c release by mitochondria. Produced cytochrome c bind to apoptosis-protease activating factor 1 (Apaf1) and result in the generation of the apoptosome. Eventually, a caspase cascade is triggered leading to apoptosis. 3. Tumor cells increase endothelial cell proliferation and vasopermeability and alter gene expression *via* vascular endothelial growth factor (VEGF) pathway. Ensuing angiogenesis facilitates tumor cell proliferation. MTAs cut off tumor blood supply by destroying its vasculature. 4. Increase in p53 concentration stimulates the production of p27 that inhibits cyclin-dependent kinase (Cdks) and thus prevents cell cycle transition at several checkpoints. P53 can also interact with some members of the Bcl-2 family and induce apoptosis *via* the aforementioned mechanism.

MTAs alter the normal structure and function of microtubules that subsequently affects microtubule assembly and spindles formation (**Figure 3**). Mitotic spindles lose the pulling power required to separate sister chromatids and cannot properly orientate them, which completely stops the process of cell division. The two-way separation of sister chromatids is regulated by SAC. Chromosome segregation does not occur until the “correct” checkpoint is determined, which is defined as a proper and stable kinetochore-microtubule attachment (94). To ensure that anaphase does not start when kinetochores are not attached or attached improperly, checkpoint proteins are recruited on kinetochores and form a mitotic checkpoint complex (MCC). This complex inhibits anaphase-promoting complex/cyclosome (APC/C), preventing degradation of securin and cyclin proteins and thus delaying anaphase onset. Errors in the test point of the cell cycle of tumor cells may result in drug sensitivity differences due to changes in the structure or expression of test point kinase (95).

As mentioned earlier, MTAs induce cell cycle arrest in the G2/M phase. Paclitaxel can block the cell cycle *via* two following mechanisms: regulation of expression of cyclin B1 and cyclin-dependent kinase (Cdk). CA-4 blocks the cell cycle in the G2/M phase by regulating the expression level of Cdc2 (96).

### Induce Apoptosis and Autophagy

Cell cycle arrest has long been known to trigger apoptosis. Apoptosis induction occurs *via* different pathways, such as phosphorylation of Bcl-2 and Bcl-xL, activation and upregulation of E2F1—all of which can instigate the release of cytochrome c (31). Activation of mammalian target of rapamycin (mTOR) is also implicated with microtubules. Interference with microtubule activity interrupts the AKT/mTOR signaling pathway, leading to hindered tumor cell proliferation *via* autophagy induction. This independent mechanism represents a unique tool for inducing mitotic arrest (97).

### Anti-Angiogenesis and Vascular Destruction

Folkman et al. postulated that tumor neovascularization is involved in tumor development and metastases (98). Destruction of tumor vasculature, starvation of tumor cells, and other strategies have been widely adopted in many types of cancer. Several cancers in mice were found to be inhibited after feeding with natural vascular inhibitors (95).

The application of MTAs becomes a new direction in the research of anti-tumor drugs. Drugs, such as paclitaxel, vinblastine, and colchicine, have potential anti-angiogenic effects on PC. Vinblastine was shown to exhibit dose-dependent anti-angiogenic activity in a chick embryo chorioallantoic membrane model. A research confirmed that tumor blood vessels could be selectively destroyed within 6 h after administration of CA-4 in an alive rat model. Notably, the effect on the intratumor blood vessels is stronger than on the extratumor ones. Some researchers are currently investigating the effects of MTAs on vascular destruction and conducting corresponding clinical trials. More than 10 types of tumor vasculature-targeting drugs are enrolled in clinical trials. Most of them act on the colchicine site in PC and advanced solid tumors.

Only a few studies have studied the side effects of MTAs such as cardiotoxicity and gastrointestinal adverse reactions. It is still unclear as to how MTAs exactly inhibit tumor angiogenesis or destroy tumor blood vessels. Vascularization requires the proliferation and transport of vascular endothelial cells, both of which are very sensitive to MTAs. Certain MTAs are speculated to affect the development of tumor blood vessels by altering the expression of vascular endothelial growth factor (VEGF). Colchicine, nocodazole, vinblastine, and vincristine were shown to reduce the production of human umbilical vein endothelial cells (HUVEC) and expression of VEGF (56, 99).

Finally, damage to tumor vasculature inflicted by MTAs is more reversible than inhibiting cell proliferation. Drugs that cause depolymerization for a short time are more suitable to serve as vascular inhibitors, whereas long-term anti-mitotic agents act as tumor cell proliferation inhibitors (100).

### p53

The core mechanism of paclitaxel affecting mitosis of cancer cells is still under investigation. Some studies proved that the inhibitory action of paclitaxel lies in alterations of microtubule transport that lead to decreased activation and translocation of androgen receptors. In addition, paclitaxel can stimulate the production of tumor suppressor protein, p53, and increase its quantity in the nucleus (101). The loss of p53 function due to defective genome unleashes the build-up of tumorigenic mutations in the cell and increases cancer cell survival. p53 signaling is associated with microtubule dynamics and expression of various tubulin isotypes (31).

## Conclusion

Tubulin is not the most optimal target for cancer targeting drugs as it requires high selectivity of agents. This in turn makes drug development relatively complex. However, current researches give hope that the creation of such agents is possible, and prolonging the survival rate in PC may not be unachievable. Humanity has long needed it.

## Author Contributions

MH—conceptualization, formal analysis, investigation, and writing (original draft). BA—writing (original draft, review, and editing) and visualization. WW—supervision, project administration, and funding acquisition. All authors contributed to the article and approved the submitted version.

## Funding

This work was supported by the National Natural Science Foundation of China (81572307 and 81773096) and by the Major Project of the Medical and Health Technology Development Program in Zhejiang Province (7211902).

## Conflict of Interest

The authors declare that the research was conducted in the absence of any commercial or financial relationships that could be construed as a potential conflict of interest.

## Abbreviations

PDAC, pancreatic ductal adenocarcinoma; PC, pancreatic cancer; GTP, guanosine triphosphate; MAP, microtubule-associated protein; HPDE, human pancreatic duct epithelium; MTAs, microtubule-targeting agents; MDAs, microtubule-destabilizing agents; MSAs, microtubule-stabilizing agents; NSCLC, non-small cell lung carcinoma; CA-4, Combretastatin-A4; SPARC, secreted protein acidic and rich in cysteine; SAC, spindle assembly checkpoint; MCC, mitotic checkpoint complex; APC/C, anaphase-promoting complex/cyclosome; Cdks, cyclin-dependent kinases; mTOR, mammalian target of rapamycin; VEGF, vascular endothelial growth factor.
