# Corrigendum: The role of microtubules in pancreatic cancer: therapeutic progress

**DOI:** 10.3389/fonc.2024.1414456

**Published:** 2024-05-01

**Authors:** Mugahed Abdullah Hasan Albahde, Bulat Abdrakhimov, Guo-Qi Li, Xiaohu Zhou, Dongkai Zhou, Hao Xu, Huixiao Qian, Weilin Wang

**Affiliations:** ^1^ Department of Hepatobiliary and Pancreatic Surgery, The Second Affiliated Hospital, School of Medicine, Zhejiang University, Hangzhou, China; ^2^ Key Laboratory of Precision Diagnosis and Treatment for Hepatobiliary and Pancreatic Tumor of Zhejiang Province, Hangzhou, China; ^3^ Research Center of Diagnosis and Treatment Technology for Hepatocellular Carcinoma of Zhejiang Province, Hangzhou, China; ^4^ Clinical Medicine Innovation Center of Precision Diagnosis and Treatment for Hepatobiliary and Pancreatic Disease of Zhejiang University, Hangzhou, China; ^5^ Department of Cardiovascular Surgery, Renmin Hospital of Wuhan University, Wuhan, China; ^6^ Clinical Research Center of Hepatobiliary and Pancreatic Diseases of Zhejiang Province, Hangzhou, China

**Keywords:** pancreatic ductal adenocarcinoma, microtubule-targeting agents, microtubules, pancreatic cancer, drugs

In the published article, there was an error in the affiliation of Bulat Abdrakhimov. Instead of “Clinical Medicine Innovation Center of Precision Diagnosis and Treatment for Hepatobiliary and Pancreatic Disease of Zhejiang University, Hangzhou, China” (affiliation #4), it should be “Department of Cardiovascular Surgery, Renmin Hospital of Wuhan University” (affiliation #5).

The authors apologize for this error and state that this does not change the scientific conclusions of the article in any way. The original article has been updated.

